# High transmission of endemic human coronaviruses before and during the COVID-19 pandemic in adolescents in Cebu, Philippines

**DOI:** 10.21203/rs.3.rs-3581033/v1

**Published:** 2023-11-16

**Authors:** Janet O. Joseph, Michelle Ylade, Jedas Veronica Daag, Rosemary Aogo, Maria Vinna Crisostomo, Patrick Mpingabo, Lakshmanane Premkumar, Jacqueline Deen, Leah Katzelnick

**Affiliations:** National Institutes of Health; National Institutes of Health, University of the Philippines-Manila; National Institutes of Health, University of the Philippines-Manila; National Institutes of Health; National Institutes of Health, University of the Philippines-Manila; National Institutes of Health; University of North Carolina School of Medicine; National Institutes of Health, University of the Philippines-Manila; National Institutes of Health

**Keywords:** Endemic coronaviruses, seroprevalence, Philippines, COVID-19, SARS-CoV-2, OC43, HKU1, 229E, NL63

## Abstract

**Background.:**

SARS-CoV-2, the causative agent of COVID-19, is a betacoronavirus belonging to the same genus as endemic human coronaviruses (hCoVs) OC43 and HKU1 and is distinct from alpha hCoVs 229E and NL63. In a study of adolescents in the Philippines, we evaluated the seroprevalence to hCoVs, whether pre-pandemic hCoV immunity modulated subsequent risk of SARS-CoV-2 infection, and if SARS-CoV-2 infection affected the transmission of the hCoVs.

**Methods.:**

From 499 samples collected in 2021 and screened by SARS-CoV-2 receptor binding domain (RBD) enzyme-linked immunosorbent assay (ELISA), we randomly selected 59 SARS-CoV-2 negative and 61 positive individuals for further serological evaluation. We measured RBD and spike antibodies to the four hCoVs and SARS-CoV-2 by ELISA in samples from the same participants collected pre-pandemic (2018–2019) and mid-pandemic (2021), before COVID-19 vaccination.

**Results.:**

We observed over 72% seropositivity to the four hCoVs pre-pandemic. Binding antibodies increased with age to 229E and OC43, suggesting endemic circulation, while immunity was flat across ages for HKU1 and NL63. During the COVID-19 pandemic, antibody level increased significantly to the RBDs of OC43, NL63, and 229E and spikes of all four hCoVs in both SARS-CoV-2 negative and positive adolescents. Those aged 12–15 years old in 2021 had higher antibodies to RBD and spike of OC43, NL63, and 229E than adolescents the same age in 2019, further demonstrating intense transmission of the hCoVs during the pandemic.

**Conclusions.:**

We observe a limited impact of the COVID-19 pandemic on endemic hCoV transmission. This study provides insight into co-circulation of hCoVs and SARS-CoV-2.

## BACKGROUND

During the past two decades there have been three major coronavirus spillovers. The severe acute respiratory syndrome coronavirus 1 (SARS-CoV-1) pandemic from 2002 to 2004 and repeated Middle East respiratory syndrome coronavirus (MERS-CoV) outbreaks since 2012 were brought under control by mitigation measures. A large number of SARS-related coronaviruses have been discovered in bats, their natural reservoir host, and SARS-CoV-2 emerged on December 31, 2019 [1] resulting in over 770 million cases and nearly 7 million deaths globally [2]. Lockdowns and other measures were implemented in the spring of 2020 and highly effective vaccines were rapidly developed and deployed starting in late 2020. Despite these efforts, SARS-CoV-2 will likely continue to circulate endemically in human populations, undergoing antigenic evolution in different regions with variants emerging under immune selective pressure [3].

Long before the emergence of SARS-CoV-1 and - 2 and MERS-CoV, four human coronaviruses (hCoVs) have circulated endemically. These include two betacoronaviruses, OC43 and HKU1, which are of the same genus as SARS-CoV-1, -2, and MERS-CoV, and two alphacoronaviruses, 229E and NL63. The average age of first infection with OC43, HKU1, 229E, and NL63 is 5 years [4], and individuals are frequently reinfected with the same virus over the course of their lives [5–7]. HCoV infection usually results in the common cold, characterized by coryza, sore throat, headache, fever, and cough [8]. The four hCoVs are estimated to have emerged between 50 and 700 years ago [9, 10]. Like SARS-CoV-1 [11] and SARS-CoV-2 [12], hCoVs such as 229E evolve antigenically, escaping immunity induced against earlier circulating strains [13]. Multiple distinct lineages circulate, with two known lineages each of HKU1 and OC43. HCoVs OC43, HKU1, 229E, and NL63 are thought to be endemic globally, although variation has been described in seroprevalence between the Americas, Africa, and Europe [14, 15].

Given the recent SARS-CoV-1 and - 2 and MERS-CoV spillovers, why are there not more circulating endemic hCoVs? It has been suggested that existing population immunity could restrict entrance of novel coronaviruses [10]. Numerous early studies during the COVID-19 pandemic explored whether hCoVs modulate SARS-CoV-2 infection and disease risk and explain reduced severity of COVID-19 in children compared to adults [16]. There are cross-reactive epitopes between the hCoVs and SARS-CoV-2 spike, especially in the S2 segment [16, 17]. Studies in adults and mice showed minimal effect of prior hCoV infection on SARS-CoV-2 infection risk [18, 19], although recent infection may protect against a COVID-19 case [20, 21]. However, pre-pandemic samples from children and adolescents were found to have higher levels of cross-neutralizing SARS-CoV-2 antibodies than older individuals [22, 23], although not in all studies [24]. Children also have higher pre-pandemic IgM responses to SARS-CoV-2 and other hCoVs, whereas antibodies to these antigens in the elderly were predominantly IgA or IgG [18, 25]. Additionally, young adults have been observed to have OC43- or NL63-induced IFN-gamma secreting T cells that cross-react with SARS-CoV-2, while older adults do not [26]. This pattern has not been observed for influenza or RSV, suggesting the effect may be coronavirus specific [26]. Together, these data suggest that adolescents compared with other age groups may be more likely to have levels of endemic hCoV immunity that could restrict SARS-CoV-2 infection, if such protection exists.

As COVID-19 transitions to an endemic disease [27], a second question is whether SARS-CoV-2 might drive other hCoVs to extinction. Each new emerging SARS-CoV-2 variant is more transmissible [28, 29], making it plausible that SARS-CoV-2 may outcompete existing hCoVs. The control restrictions put in place during the COVID-19 pandemic resulted in the lowest reported incidence of common respiratory illnesses, such as influenza A and B viruses as well as RSV disease [30]. While most of these viruses rebounded once restrictions were lifted, some lineages may have gone extinct while restrictions were in place. For instance, it has been proposed that the pandemic dramatically reduced circulation of the Influenza B/Yamagata lineage, one of the two major influenza B lineages to circulate globally prior to the pandemic [31, 32], because it had a lower basic reproduction number. Whether such competition exists between SARS-CoV-2 and the hCoVs remains to be fully elucidated. A modeling study explored different scenarios of cross-protection between SARS-CoV-2 and endemic coronaviruses and found that intermediate cross-protection between viruses would lead to less frequent but larger hCoV epidemics while high cross-protection might eliminate hCoV transmission, with SARS-CoV-2 outcompeting the hCoVs [7].

Since 2017, we have followed a cohort of adolescents in the Cebu province of the Philippines. The first confirmed COVID-19 case in the Philippines was reported on January 30, 2020 and the government implemented a pandemic mitigation strategy consisting of lockdowns, quarantine, COVID-19 testing, hospital control, and economic relief starting in March 2020. Over 4 million cases and 66,000 deaths have been documented nationwide in outbreaks occurring in several waves [33]. Epidemics in Cebu Province mirrored those seen nationwide. Mass COVID-19 vaccination was started in adults in March 2021 and adolescents in November 2021. The objectives of this study were to: (1) assess the seroprevalence and level of immunity to the four hCoVs in Cebu, Philippines before and during the COVID-19 pandemic but prior to vaccination, (2) test if differences in immunity to the hCoVs were associated with the odds of infection with SARS-CoV-2, and (3) evaluate the level of boosting to hCoVs after infection with SARS-CoV-2.

## METHODS

### Ethics Statement

This study was reviewed and approved by the University of the Philippines – Manila Research Ethics Board (UPM-REB, Protocol Code: 2020-773-01). The cohort was embedded within a larger, longitudinal dengue study (clinicaltrials.gov number: NCT03465254). Parents or guardians of all participants provided written informed consent. Verbal assent was obtained from the participants and documented. Standards for the Reporting of Diagnostic Accuracy Studies (STARD) guidelines were followed for all components of the study [34]. A positive control serum was collected following informed consent on NIH Institutional Review Board-approved study NCT01306084. A negative control serum from an African green monkey was collected on NIAID Animal Care and Use Committee approved protocol 14DEN34, parent protocol NIAID ASP LID 9.

### Study Site and Participants

In 2017, we enrolled 2,996 healthy participants 9 to 14 years of age in Bogo and Balamban, Cebu province, Philippines. Serum samples were collected from all participants at baseline and approximately every year thereafter. The sera were processed, aliquoted and stored in −80°C prior to testing. From June to October 2021 (during the third wave of COVID-19) we obtained sera from 1403/2,996 (47%) of participants. We randomly selected a subset of 499/1403 (36%) serum samples and their paired pre-pandemic samples for SARS-CoV-2 serologic testing. In the 2021 samples, approximately a third of the cohort had seropositivity to SARS-CoV-2, as described in a separate manuscript (in preparation).

We used a case-control study design to randomly select n = 60 serum samples that were SARS-CoV-2 seropositive and n = 60 that were seronegative in 2021 for in-depth serological testing. With 60 participants per group, we have 80% power to detect a difference of 0.26 in ELISA OD between groups, assuming a standard deviation of 0.5 at a significance level of 0.05. During sample selection, one sample was accidentally replaced with another, leading to selection of n = 59 SARS-CoV-2 RBD negative and n = 61 SARS-CoV-2 RBD positive individuals for further study. We also tested paired pre-pandemic samples from the same 120 participants collected in 2018 or 2019.

### Enzyme-linked immunosorbent assay (ELISA)

We used ELISAs to measure the seropositivity to the four hCoV and SARS-CoV-2 antigens. Recombinant RBDs for SARS-CoV-2 (GenBank accession number: QIS60558.1), and prototype strains for 229E (P15423.1), NL63 (Q6Q1S2.1), OC43 (P36334.1), and HKU1 (Q0ZME7.1) were produced and provided by L. Premkumar as described previously [35]. Full length recombinant soluble spike trimer proteins for Wuhan SARS-CoV-2 (SARS-CoV-2 S-2P(15–1208)-T4f-3C-His8-Strep2×2), 229E (HCoV-229E S-2P(17–1108)-T4f-3C-His8-Strep2×2), NL63 (HCoV-NL63 S-2P(16–1291)-T4f-3C-His8-Strep2×2), OC43 (HCoV-OC43 S-2P-3C-His8-Strep2×2), and HKU1 (HCoV-HKU1 S-2P(14–1276)-T4f-3C-His8-Strep2×2) were produced as described previously by the Frederick National Laboratory for Cancer Research [36].

ELISAs were performed as described previously [35]. Each antigen was tested separately. Microtiter plates (Greiner High Binding Plates, Cat #: GBO 655061) were coated with 50 μL of antigen diluted in 1X tris-buffered saline (TBS) to a concentration of 4 μg/ml and incubated for one hour at 37°C on a shaker at 40rpm. Coating was discarded and plates were blocked with 3% non-fat dry milk (Apex Bioresearch Products, Cat #: 20–241) in 0.05% TBST (Tween 20, Sigma-Aldrich, Cat #: P7949) and incubated for one hour at 37°C. Blocking buffer was removed, and 50 μL of heat inactivated sera prepared at a 1:40 dilution in blocking solution were added to plates in duplicate and incubated for 1 hour at 37°C. A 1:2500 dilution of secondary antibody was prepared by adding 3 μL each of alkaline phosphatase conjugated goat anti-human IgA (abcam, Cat #: ab97212), IgG (Sigma-Aldrich, Cat #: A9544), and IgM (Sigma-Aldrich, Cat #: A3437) into 7.5 mL of blocking solution. Plates were washed with 0.2% TBST using the Agilent-BioTek 405 TS microplate washer, 50 μL of secondary antibody solution was aliquoted into each well, and the plates were wrapped with aluminum foil and incubated for 1 hour at 37°C. Substrate solution was prepared by adding SIGMAFAST(TM) p-Nitrophenyl phosphate set (Sigma-Aldrich, Cat #: N2770-50SET) into 20 mL di-water in a 50 μL amber tube (Greiner Cat #: 82051–628). The mixture was gently inverted on the bottle roller for 30 minutes. Plates were washed with 0.2% TBST and 50 μl of substrate solution was added into each well and incubated at room temperature for 10 minutes. The reaction was stopped by the addition of 25 μl of 1N NaOH, and absorbances were measured at 405 nm on the BioTek Cytation7 microplate reader. A positive control from an individual determined to have reactivity to all endemic hCoVs and SARS-CoV-2 was included on each plate. Each plate also contained a negative control consisting of serum from an African green monkey detectable by the secondary anti-human IgG antibody but negative to all endemic hCoVs and SARS-CoV-2. Monkeys are not known to be natural hosts for hCoVs and this monkey was observed in an enclosed facility and presumed to not have been contaminated with hCoVs.

### Data processing

A previous study of this assay used positive and negative control samples and a receiver operator curve to identify a definition of positivity as greater than OD 0.265, which yielded a sensitivity of 97.7% and specificity of 100% [35]. In our study, negative control samples had an average OD of 0.24 (SD: 0.07) in RBD assays and 0.25 (SD: 0.08) in spike assays, consistent with Premkumar et al. Thus, all ELISA values were corrected by subtracting the average of the negative control on the matched plate to account for plate-to-plate and inter-day assay variability. For RBD and spike assays, within-experiment mean difference between replicates ranged from − 0.02 to 0.04 (range of standard deviation, SD: 0.02 to 0.14), with strong correlation between replicates (range r^2^: 0.93–0.99) (**Fig. S1**). We defined a rise in antibodies as a change in ELISA OD > 0.2, which was greater than the standard deviation of between-experiment differences (mean 0.04, SD: 0.17) and 3 times the standard deviation of the negative controls.

### Statistical analysis

All analyses were performed in R 4.0.2 (R Foundation for Statistical Computing) and GraphPad Prism. Power calculations were estimated using the stats package in R (power.t.test and power.prop.test). Data were visualized using the ggplot2 package. Differences between groups by demographic variables were evaluated with the Welch two-sample t-test (continuous variables) or Pearson’s Chi-squared test (proportions) using the gtsummary package. Odds ratios were estimated using unadjusted and adjusted logistic regression models (binomial with a logit link) using the glm function and plotted using the forestplot package. Linear regression was performed using the lm function in the stats package. Changes in antibody ELISA ODs between years were estimated using paired t-tests. Pearson’s correlation coefficients were visualized using the corrplot package.

## RESULTS

Our study characterized hCoV immunity in 120 adolescents in Cebu, Philippines who were SARS-CoV-2 seropositive (n = 61) and seronegative (n = 59) in 2021 (Table 1) We found no significant differences in demographic characteristics between these groups.

### Seropositivity to the endemic hCoVs was high in the Philippines before the COVID-19 pandemic.

**Figure 1.**
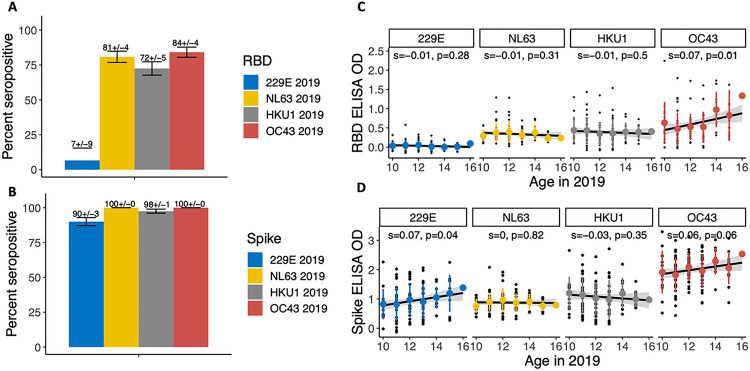
Seroprevalence to the four hCoVs before the COVID-19 pandemic. Bar chart (proportion with standard errors) showing seroprevalence to the four endemic CoVs as measured by **(A)**RBD and **(B)** spike. Positivity was defined as ELISA OD values more than 3 times the standard deviation of the negative controls (OD >0.2). ELISA OD by age in 2019, stratified by antigen for **(C)** RBD and **(D)** spike ELISAs. Colored points and lines show means and data ranges, black lines show linear relationships, with grey shapes indicating the 95% confidence interval. The relationship between age and ELISA OD was estimated with linear regression; slope estimates and p-values are shown.

We first measured antibodies to hCoVs in sera collected in years 2018/2019 using the RBD ELISA, which is used for specific detection of past exposure with a given hCoV. In this population, 72–84% of adolescents were seropositive by RBD to NL63, HKU1, and OC43, while seropositivity to 229E was lower (Fig. 1A). When measured by spike antigen, the proportion seropositive was high for all four hCoVs (90–100%, Fig. 1B). Variation was observed in antibody binding to RBD and spike for each antigen. We found ELISA ODs increased with age for 229E spike and OC43 RBD (with a trend for OC43 spike) (Fig. 1C–D), while immunity was flat across ages for HKU1 and NL63.

### Prior hCoV immunity was minimally associated with SARS-CoV-2 infection risk.

**Figure 2.**
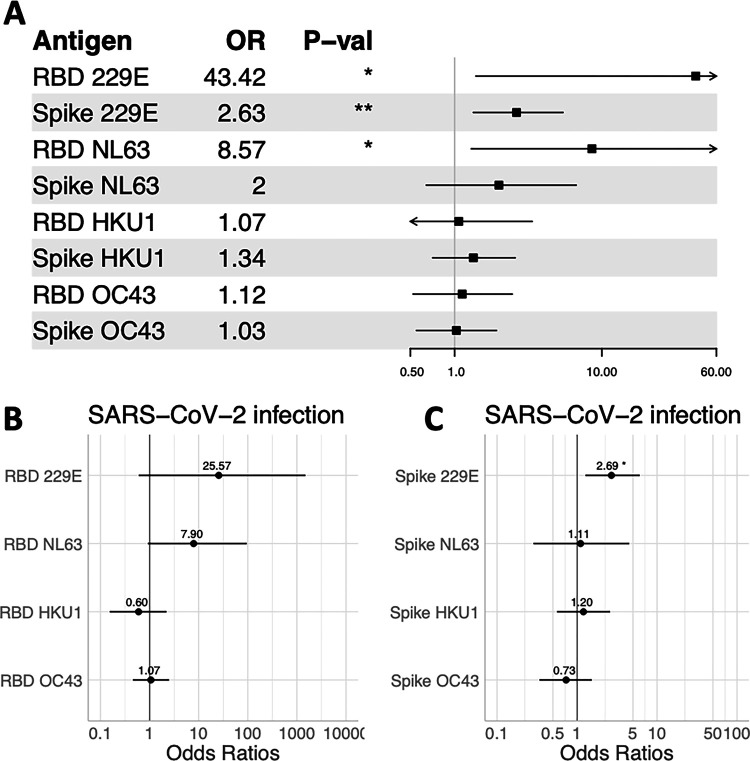
Odds of SARS-CoV-2 infection by endemic hCoV ELISA OD prior to the COVID-19 pandemic. **(A)** Forest plot of the odds ratios from unadjusted logistic regression of the effect of baseline ELISA OD as measured by RBD or spike on SARS-COV-2 infection. Models adjusted by age, sex, and study site are show in **Fig. S2**. Odds ratios for SARS-CoV-2 infection, where all measures of pre-COVID-19 antibodies to endemic CoVs were included in the same model, are shown for **(B)** RBD and **(C)** spike.

We next tested whether antibodies to the hCoVs prior to the COVID-19 pandemic were associated with risk of SARS-CoV-2 infection. We did not observe an association between positivity to betacoronaviruses OC43 and HKU1 and SARS-CoV-2 infection (Fig. 2A). However, high levels of antibodies to alphacoronaviruses 229E and NL63 were associated with increased odds of SARS-CoV-2 infection (Fig. 2A), even after adjusting for age, sex, and study site (**Fig. S2**). When we included all RBD (Fig. 2B) or spike hCoV (Fig. 2C) antibody measures in the same logistic models, only the effect of 229E spike antibodies remained a significant risk factor for SARS-CoV-2 infection.

### High endemic hCoV transmission was observed during the COVID-19 pandemic.

**Figure 3.**
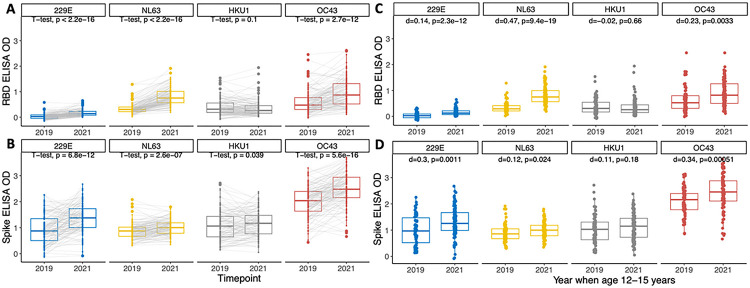
Change in antibodies to RBDs and spikes of hCoVs from before to during the COVID-19 pandemic. Comparison of 2019 vs. 2021 ELISA OD values to each RBD **(A)** and spike antigen **(B)**. ELISA OD values between years were compared with a paired t-test. The difference (d) in ELISA values to each RBD **(C)** and spike antigen **(D)** were also compared for adolescents age 12–15 years in 2019 vs. 2021 using a linear model, adjusted for age to account for differences in age distributions between years.

We then evaluated whether the endemic hCoVs circulated during the COVID-19 pandemic. Between 2019 and 2021, we observed significant increases in antibodies to the RBDs of OC43, NL63, and 229E and spikes of all four hCoVs (Fig. 3A–B). Notably, adolescents ages 12–15 years old in 2021 had higher antibodies to RBDs and spikes of OC43, NL63, and 229E than adolescents the same age in 2019, even after adjusting for differences in age distributions between the groups (Fig. 3C–D). We observed little change in HKU1 antibodies.

### SARS-CoV-2 infection is not associated with reduced endemic hCoV infection.

**Figure 4.**
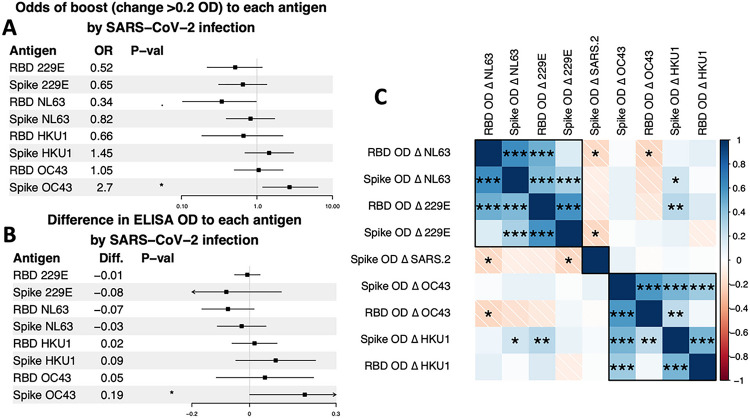
Changes (Δ) in antibodies to RBDs and spikes of hCoVs between 2019 and 2021 in those with vs. without SARS-CoV-2 infection. **(A)** Odds of experiencing a boost (change of >0.2 OD) to each antigen by SARS-CoV-2 infection. Odds ratios were estimated using logistic regression. **(B)**Magnitude of change in ELISA OD to each antigen by SARS-CoV-2 infection. Differences were estimated using linear regression, with ELISA ODs in 2021 modeled as a function of SARS-CoV-2 infection and ELISA ODs in 2019. **(C)** Pearson’s correlation coefficients among changes in ELISA ODs of the hCoV antigens. Colors indicate correlation, stars indicate significance: <0.05, *; <0.01, **; <0.001, ***.

Finally, we tested whether SARS-CoV-2 infection was associated with the odds of infection with hCoVs. There were no differences in boost (> 0.2 rise in RBD OD, Fig. 4A) or any significant change in ELISA OD (Fig. 4B) to the four hCoV RBDs by SARS-CoV-2 infection. The only significant effect was that SARS-CoV-2 infection was associated with a significant rise in antibodies to the spike of OC43. When we changed our definition of SARS-CoV-2 infection to include those with a large rise (OD rise > 0.6, equivalent to a 4-fold rise in titers) in SARS-CoV-2 spike antibodies, with or without an increase in SARS-CoV-2 RBD antibodies (**Fig. S3**), SARS-CoV-2 infection was also associated with a significant rise in antibodies to the spike of HKU1 and a decrease in antibodies to NL63 RBD. We also evaluated how changes in antibodies between years 2018/2019 and 2021 correlated among the five hCoVs. The strongest correlations were observed between 229E and NL63 and between OC43 and HKU1 (Fig. 4C). The magnitude of change in SARS-CoV-2 spike antibodies was not directly correlated with the betacoronavirus spike antibodies but was associated with a modest decline in alphacoronavirus spike antibodies.

## DISCUSSION

In this study, we observed high levels of endemic hCoV circulation before and during the COVID-19 pandemic both in adolescents with and without SARS-CoV-2 infections. Pre-pandemic hCoV immunity had minimal effect on odds of SARS-CoV-2 infection and similarly, SARS-CoV-2 infection did not appear to modify the odds of infection with any of the hCoVs. These findings suggest that transmission of the endemic hCoVs in this population continued during the COVID-19 pandemic.

We observed high seroprevalence to the four endemic hCoVs in adolescents in the Philippines before the COVID-19 pandemic, suggesting intense circulation in this population. A recent study also showed young children in Biliran, Philippines hospitalized with severe respiratory infections before the COVID-19 pandemic had high seroprevalence to all four hCoVs, indicating a high attack rate [37]. Previous studies in the USA, Germany, Hong Kong, and China have observed high seroprevalence to the hCoVs in children over age 10 (over 50%), with higher levels to OC43 and 229E, and similar levels to those observed in older adults [4]. A pre-pandemic seroprevalence and seroincidence study in Beijing showed that IgG levels are high across hCoVs by age 4–6 years, peaking at ages 4–6 or 7–14 and then dropping to lower positivity rates for older ages. IgM positivity, a measure of recent exposure, was not observed in those over age 14, suggesting most infections occur by age 14 and possible waning of immunity for older ages [38]. A study of seroincidence to OC43 and 229E also observed higher seroincidence in children less than 14 compared to adults [39]. Notably, the magnitude of antibodies to 229E RBD in our study was lower than expected. However, we used the prototype 229E RBD, which is from a strain isolated before 1990, and previous work has shown strong antigenic evolution of 229E since that time [13]. Given that we observed high prevalence and increasing immunity with age to 229E spike, which may contain more conserved epitopes, the low prevalence to 229E may be due to the specific antigen strain used and, therefore, may not accurately reflect the prevalence in our population. This finding highlights the complexity of measuring immunity to coronaviruses, as the selection of matched antigens to the population may be important for accurate measurement of seroprevalence.

While previous studies found that adolescents were more likely to have cross-neutralizing antibodies to SARS-CoV-2, we did not find that baseline immunity to the betacoronaviruses was associated with protection against SARS-CoV-2 infection. Instead spike antibodies to 229E were associated with increased SARS-CoV-2 infection. Pre-pandemic neutralization and ACE2 binding inhibition has been associated with both OC43 and NL63 spike activity, suggesting cross-immunity may not be limited to the betacoronaviruses [23, 40]. However, a previous study showed that mice were not protected against SARS-CoV-2 challenge by vaccination with hCoVs [19]. Given limited cross-reactivity between 229E and SARS-CoV-2 and the direction of the effect, our observation may indicate that 229E spike antibodies are markers for a higher overall risk for coronavirus infection in this population.

Our most surprising finding was the high circulation of hCoVs during the pandemic, especially OC43, NL63, and 229E. The observation that adolescents in 2021 had higher antibodies to OC43, NL63, and 229E than adolescents of the same age in 2019, with adjustment for covariates, suggests that transmission of these hCoVs was higher during the COVID-19 pandemic than in the period immediately preceding the pandemic. This finding further suggests the COVID-19 pandemic had a limited effect on the circulation of hCoVs in an adolescent population. Notably, we found minimal change in HKU1 RBD antibodies, despite relatively high levels of immunity in the population pre-pandemic, suggesting more limited circulation of this virus. This observation may be due to limited circulation of HKU1 in this population in general, or to a reduction in transmission of HKU1 during the pandemic. HKU1 infection less common globally, may be modified by immunity to OC43 [5, 41], and may induce more mild illness and a reduced antibody response [42]. Whether transmission of HKU1 declined in a manner like Influenza B/Yamagata [31, 32] because of the pandemic should be studied in other populations.

Additionally, our data do not support the hypothesis that SARS-CoV-2 induces immunity that prevents infection with other hCoVs. Overall, we see similar odds of infection as measured by RBD with betacoronaviruses and alphacoronaviruses in those with and without SARS-CoV-2 infection. Previous studies have shown antibodies to endemic hCoVs, especially betacoronaviruses, are reactivated during SARS-CoV-2 infection [18, 43, 44]. SARS-CoV-2 mRNA vaccination can also boost hCoV immunity [45]. We also found that those with SARS-CoV-2 infections were slightly more likely to experience a boost in antibodies to the spikes of OC43 and to a lesser extent HKU1, suggesting SARS-CoV-2 infection expands cross-reactive antibodies targeting shared epitopes on the betacoronavirus spike proteins. Notably, in our study, the magnitude of change in antibodies to SARS-CoV-2 did not correlate with either betacoronavirus, potentially because the dominant epitopes targeted on SARS-CoV-2 are in regions that are not cross-reactive with the betacoronaviruses due to differences in sequence homology [16].

Our study has several limitations. The RBD antigen used for 229E performed poorly at measuring immunity in this population. Updating the antigen to a more recent strain could increase the accuracy of measures of seroprevalence. Further, to enable the testing of numerous antigens, we were limited in the number of samples we could test. This may have limited our power to detect subtle protective effects.

## CONCLUSIONS

Overall, our study demonstrates high hCoV circulation in an adolescent population in the Philippines, both before and during the COVID-19 pandemic. The pandemic appears to have had little effect on the circulation of hCoVs, except possibly HKU1. Our study demonstrates how a new emerging coronavirus can become an endemic pathogen, joining the other hCoVs in causing annual epidemics in populations around the world.

## Figures and Tables

**Table 1.** Comparison of baseline characteristics among adolescents who were SARS-CoV-2 positive vs. negative, as measured by RBD ELISA.

Characteristic	NEGATIVE, N = 59^1^	POSITIVE, N = 61^1^	p-value^2^
Age	12 (2)	12 (1)	0.2
Sex			0.6
F	35 / 59 (59%)	33 / 61 (54%)	
M	24 / 59 (41%)	28 / 61 (46%)	
Site			0.5
Balamban	33 / 59 (56%)	30 / 61 (49%)	
Bogo	26 / 59 (44%)	31 / 61 (51%)	

1 Mean (SD); n / N (%)

2 Welch Two Sample t-test; Pearson’s Chi-squared test

## Data Availability

The data sources and programs used for analyses are detailed in the Methods section. The code and individual-level associated data will be made available on Zenodo at the time of publication. All materials are covered under standard material transfer agreements. Inquiries for data or materials should be addressed to Leah Katzelnick (leah.katzelnick@nih.gov) and Jacqueline Deen (deen.jacqueline@gmail.com).

## List Of Abbreviations

hCoVs: Human coronaviruses
RBD: Receptor binding domain
ELISA: Enzyme linked immunosorbent assay
SARS-CoV-2: Severe acute respiratory syndrome coronavirus 2
SARS-CoV-1: Severe acute respiratory syndrome coronavirus 1
MERS-CoV: Middle East respiratory syndrome coronavirus
COVID-19: Coronavirus disease 2019
